# Speed Consistency in the Smart Tachograph †

**DOI:** 10.3390/s18051583

**Published:** 2018-05-16

**Authors:** Daniele Borio, Eduardo Cano, Gianmarco Baldini

**Affiliations:** European Commission, Joint Research Centre (JRC) Directorate for Space, Security and Migration, Via Enrico Fermi 2749, 21027 Ispra (VA), Italy; eduardo.cano-pons@publications.europa.eu (E.C.); gianmarco.baldini@ec.europa.eu (G.B.)

**Keywords:** Global Navigation Satellite System, GNSS, motion conflict, odometer, Smart Tachograph, ST

## Abstract

In the transportation sector, safety risks can be significantly reduced by monitoring the behaviour of drivers and by discouraging possible misconducts that entail fatigue and can increase the possibility of accidents. The Smart Tachograph (ST), the new revision of the Digital Tachograph (DT), has been designed with this purpose: to verify that speed limits and compulsory rest periods are respected by drivers. In order to operate properly, the ST periodically checks the consistency of data from different sensors, which can be potentially manipulated to avoid the monitoring of the driver behaviour. In this respect, the ST regulation specifies a test procedure to detect motion conflicts originating from inconsistencies between Global Navigation Satellite System (GNSS) and odometry data. This paper provides an experimental evaluation of the speed verification procedure specified by the ST regulation. Several hours of data were collected using three vehicles and considering light urban and highway environments. The vehicles were equipped with an On-Board Diagnostics (OBD) data reader and a GPS/Galileo receiver. The tests prescribed by the regulation were implemented with specific focus on synchronization aspects. The experimental analysis also considered aspects such as the impact of tunnels and the presence of data gaps. The analysis shows that the metrics selected for the tests are resilient to data gaps, latencies between GNSS and odometry data and simplistic manipulations such as data scaling. The new ST forces an attacker to falsify data from both sensors at the same time and in a coherent way. This makes more difficult the implementation of frauds in comparison to the current version of the DT.

## 1. Introduction

The aim of the Smart Tachograph (ST), the new revision of the Digital Tachograph (DT) [1], is to improve safety in the transportation sector by recording the driving time, breaks, rest periods as well as periods of other work undertaken by a driver of commercial vehicles. Monitoring the behaviour of drivers is expected to discourage the infringement of road regulations, forcing drivers to respect rest periods and speed limits. While breaking road rules may, at least conceptually, increase productivity, a tired driver is more prone to accidents with potentially severe effects on people and infrastructures. The use of the tachograph is prescribed for vehicles having a mass of more than 3.5 tonnes (in goods transport) and carrying more than 9 people including the driver (in passenger transport) [2]. The tachograph is an electronic device, which collects data from several sensors and records driving time, breaks as well as rest periods of a driver. While tachographs are digital since 2006 [1], a second generation of DTs has been recently mandated by the European Parliament and Council [2,3]. In particular, EU regulation 165/2014 [2] adopted in February 2014 foresees the introduction of a new generation of DTs, called “Smart Tachographs”, with increased security mechanisms, a Global Navigation Satellite System (GNSS) component and different communication interfaces. The new ST is equipped with an interface with GNSS, including the European GNSS, Galileo, and the European Geostationary Navigation Overlay Service (EGNOS). Position, Velocity and Time (PVT) information obtained through a GNSS receiver is used to record daily work periods including the start and stop locations of the vehicle. In addition to the GNSS receiver, the ST is interfaced with other sensors mounted on the vehicle. For example, the ST could retrieve speed information through the vehicle On-Board Diagnostics (OBD) system. Data from different sensors are processed by the Vehicle Unit (VU), the main component of the ST, which is in charge of processing and storing the different pieces of information. The availability of data from different sensors strengthens the ST against possible data manipulation attacks. In particular, a driver may be tempted to falsify the data recorded by the tachograph, for example, to circumvent the regulations and to avoid rest periods for an economic gain. In order to reduce the risk of data falsification, regulation [3] mandates the periodic cross-validation of the data provided by the GNSS receiver and by the on-board sensors. Regulation [3] provides the technical specifications for the ST and details the role of GNSS and Galileo. A procedure is described to verify the consistency between odometry and GNSS data. In this respect, a motion conflict is generated if a significant discrepancy between GNSS and odometry information is observed. This error is recorded by the ST and will be verified by law enforcers during an inspection. GNSS receivers are vulnerable to several threats such as jamming [4], spoofing [5], meaconing [6] and data faking [7]. While jamming prevents the receiver to operate and can be easily detected by recording a long period of GNSS signal absence, the other threats mentioned above are more subtle as they involve the provision of false PVT information. In particular, they could be more difficult to detect as, apparently, the receiver is working normally. In GNSS spoofing, false Radio Frequency (RF) GNSS signals are provided to the receiver that will determine a false PVT solution. Meaconing is a simplified form of spoofing where real GNSS signals are recorded and re-broadcast at a later stage. GNSS signals can be recorded in a static scenario and provided to the receiver when the vehicle is moving. Without data cross-validation, the GNSS receiver may be forced to believe that the vehicle is static and that the driver is respecting resting periods while he/she is actually driving. GNSS data faking is obtained by replacing the data sentences provided as output by the GNSS receiver with false PVT information.

Spoofing, meaconing and data faking can be mitigated by verifying GNSS information with the data provided by the other sensors available on the vehicle. For this reason, regulation [3] prescribes to periodically compare the speed provided by independent sources of information, such as the odometer and the GNSS receiver, to trigger a “vehicle motion conflict” event (Section 3.9 of [3]). Details on the tests introduced by the ST regulation are provided in Section 3. Data comparison, as mandated by the ST regulation forces an attacker to falsify in a coherent way the data provided by both GNSS receiver and odometer. This significantly increases the complexity of an attack.

The goal of this paper is to experimentally evaluate the effectiveness of the tests mandated by the ST regulation to prevent data manipulation. In this respect, we implemented the procedure mandated by [3] (Appendix 12), which specifies the tests to be implemented to verify the consistency between GNSS and odometry data. The procedure defined by the ST regulation was designed in order to mitigate the effects of measurement outliers and of transient values and takes into account the tolerances of the sensors used for retrieving speed measurements.

The analysis has been performed using experimental data collected using three different vehicles and considering two types of scenarios: light-urban and highway environments. The vehicles were equipped with multi-constellation GNSS receivers capable of processing Galileo signals. Moreover, OBD data readers were used to retrieve the vehicle speed. GNSS position and velocity were obtained using code Single Point Positioning (SPP) [8] without external corrections. In particular, the velocity, as directly provided by the receivers, was used.

While logistic and cost reasons prevented the use of a real commercial vehicle above 3.5 tonnes, the setup and the measurement campaigns conducted are considered realistic. The two scenarios analysed cover the most common situations that can be encountered by a commercial vehicle. More than 10 h of GNSS and odometry data were collected and used for the analysis. From the analysis, it emerges that the decision statistics used for the detection of motion conflicts are resistant to synchronization errors and latencies between GNSS and odometry data. Moreover, the decision statistics are only marginally impacted by data gaps that can occur, for example, in the presence of tunnels, where the reception of GNSS signals is not possible. Depending on the metric used for motion conflict detection, data gaps up to 2.5 min are tolerated. In the experiments conducted, no false alarms were recorded confirming the effectiveness of the procedure defined by the ST regulation.

Experiments show that, with the new regulation, is not sufficient to falsify GNSS information alone and an attacker has to forge data from both the vehicle sensor and the GNSS receiver, simultaneously. The paper significantly extends the analysis conducted in [9] that considered only a light-urban scenario. The results presented here are complementary to those discussed in [9]. Moreover, the data collected in the highway scenario are new and the related results were not presented before. The analysis of the impact of tunnels and of data gaps is also new and it is one of the contributions of the paper.

The remainder of this paper is organised as follows: Section 2 introduces the signals adopted for speed verification. The decision statistics prescribed by the DT regulation are detailed in Section 3. The experimental setup with the two scenarios considered are discussed in Section 4 whereas experimental results obtained under nominal conditions are analysed in Section 5. Experimental results obtained when considering some forms of data manipulation are discussed in Section 6 and conclusions are finally drawn in Section 7.

## 2. System Overview

As described in the previous section, the ST will use the data provided by the GNSS receiver and by the vehicle odometer to periodically verify the consistency between the speeds derived from the two sensors [3]. In this respect, the basic signals used by the VU of the ST are:

- the speed provided by the odometer, $S_{odo}\left(t_{n}\right)$, and
- the speed provided by the GNSS receiver and computed using both GPS and Galileo signals.

The odometer speed, $S_{odo}\left(t_{n}\right)$, is obtained by interrogating the vehicle sensor through the OBD interface [10]. The OBD system defines a serial protocol where information is provided as a response to a data request, which usually corresponds to a Parameter IDentifier (PID). For example, speed information is retrieved using PID 13. Since the odometer speed is provided in an asynchronous way, $S_{odo}\left(t_{n}\right)$ is sampled at irregular time instants, $t_{n}$. The odometer speed is provided as an unsigned 8 bit integer. For this reason, $S_{odo}\left(t_{n}\right)$ assumes values in the [0,255] km/h range with a quantization step of 1 km/h. The sample instants, $t_{n}$, are usually defined by the vehicle on-board clock that is periodically steered to GNSS time [3].

GNSS receivers usually provide 3D PVT information. More specifically, vehicle velocity can be retrieved as a 3D vector. While the velocity vector can be determined as the time derivative of the vehicle position, more accurate velocity estimates can be obtained by using Doppler measurements that need to be derived by the receiver [8]. In this paper, we consider the use of Doppler derived velocity. The speed is then determined as the absolute value of the velocity vector defined by the three components, $V_{E}\left(t\right)$, $V_{N}\left(t\right)$ and $V_{U}\left(t\right)$:

(1) $$S_{GNSS}\left(t\right)=\sqrt{V_{E}^{2}\left(t\right)+V_{N}^{2}\left(t\right)+V_{U}^{2}\left(t\right)}.$$

$V_{E}\left(t\right)$, $V_{N}\left(t\right)$ and $V_{U}\left(t\right)$ are defined in a local East, North, Up (ENU) frame. In (1), the different components are expressed as a function of time. However, GNSS velocity is determined at discrete time instants and the quantities in (1) are regularly sampled:

(2) $$S_{GNSS}\left(nT_{s}\right)=\sqrt{V_{E}^{2}\left(nT_{s}\right)+V_{N}^{2}\left(nT_{s}\right)+V_{U}^{2}\left(nT_{s}\right)}$$

where $T_{s}$ is the sampling interval and *n* the time index defined by the local clock of the GNSS receiver. This local clock is assumed to be steered to a GNSS time. The ST regulation prescribes the usage of the National Marine Electronics Association (NMEA) 0183 protocol [3] for the data exchange between GNSS receiver and VU. In this case, $S_{GNSS}\left(nT_{s}\right)$ is provided directly as part of the NMEA *Recommended Minimum Data* (RMC) sentence.

**Figure 1:**
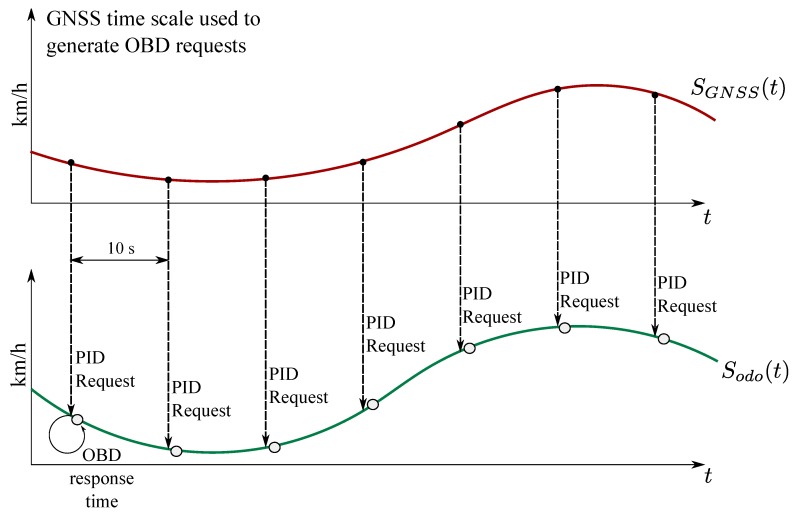
Approach adopted for the computation of the absolute speed difference. A time pulse is generated every 10 s and used to retrieve the speed from the odometer.

In order to compute the decision statistics defined by the ST and detailed in Section 3, the data from the odometer and the GNSS receiver have to be synchronized. For this reason, the procedure illustrated in Figure 1 has been adopted.

The GNSS time scale is used to generate requests that are sent through the OBD interface to retrieve $S_{odo}\left(t_{n}\right)$. ST regulation [3] foresees that speed differences between the GNSS receiver and the odometer are computed at least every 10 s. When this time interval is adopted, a speed measurement from the GNSS receiver is retained every 10 s and, at the same time, it is assumed that a PID is sent to the OBD interface to retrieve $S_{odo}\left(t_{n}\right)$. The OBD provides the speed measurement with a small response time, which introduces a small error in $S_{odo}\left(t_{n}\right)$. In particular,

(3) $$S_{odo}\left(t_{n}\right)=S_{odo}\left(nT_{s}+\Delta T\right)\approx S_{odo}\left(nT_{s}\right)+\Delta T{\dot{S}}_{odo}\left(nT_{s}\right)=S_{odo}\left(nT_{s}\right)+\eta_{syn}\left(nT_{s}\right)$$

is used for the computation of the speed differences. In (3), ΔT is the OBD response time and ${\dot{S}}_{odo}\left(nT_{s}\right)$ is the derivative of the vehicle speed at the instant $nT_{s}$. The time instant $nT_{s}$ is referred to the GNSS time scale and $T_{s}=10$ s. The term

(4) $$\eta_{syn}\left(nT_{s}\right)=\Delta T{\dot{S}}_{odo}\left(nT_{s}\right)$$

is the synchronization error affecting the speed measurements from the odometer. It is noted that a similar scheme can be obtained by considering an external timing source for the retrieval of the measurements from the two sensors.

The scheme depicted in Figure 1 represents a possible approach for roughly synchronizing the data from the two sensors in a real-time implementation. In the tests described in the rest of the paper, data were collected with two independent sensors, a GNSS receiver and an OBD data reader, which were not synchronized. The data were however collected using a high sampling rate and this allowed the emulation of the approach shown in Figure 1. In particular, the data from the two sensors were re-sampled with a sampling rate of 10 Hz. Re-sampling was performed using interpolation that was necessary to compensate for the fact that the OBD data were sampled at irregular time instants. Then, the time offset between GNSS and odometry data was determined through time series correlation. This time offset was used to align the raw datasets obtained without interpolation. Finally, the approach detailed in Figure 1 was implemented: every 10 s, a GNSS measurement was retained along with the next available odometer speed with the closest time stamp. In this way, it was possible to obtain data roughly synchronized. The effects of relative data latencies are analysed in Section 6.

Using the approach detailed above, it was finally possible to compute the speed differences:

(5) $$\Delta \bar{S}\left(nT_{s}\right)=S_{odo}\left(t_{n}\right)-S_{GNSS}\left(nT_{s}\right)=S_{odo}\left(nT_{s}\right)+\eta_{syn}\left(nT_{s}\right)-S_{GNSS}\left(nT_{s}\right)=\Delta S\left(nT_{s}\right)+\eta_{syn}\left(nT_{s}\right)$$

where symbol $\bar{\cdot }$ denotes the impact of synchronization errors and $\Delta S(nT_{s})$ is the ideal speed difference at the time instant $nT_{s}$. $\Delta \bar{S}\left(nT_{s}\right)$ is the basic signal used for the computation of the decision statistics described in the next section.

## 3. Decision Statistics

Speed differences (5) are used for the computation of the decision statistics mandated by the ST regulations for verifying the consistency between GNSS and odometry data. The absolute values of (5) are at first computed:

(6) $$R\left(nT_{s}\right)=\left|\Delta \bar{S}\left(nT_{s}\right)\right|.$$

An analysis windows is then used to select 5 min of consecutive data that are combined for the computation of the decision statistics. With a sampling interval equal to 10 s, N=30 absolute speed differences are selected. According to [3], the decision statistic

“*shall be computed as the average of 80% of the remaining values, after having eliminated the highest ones in absolute values*.”

In this way, the decision statistic is defined as the asymmetric trimmed mean of the absolute speed differences:

(7) $$M_{T}\left(nT_{s}\right)=\frac{1}{N_{80}}\sum_{i=0}^{N_{80}-1}R_{\left(i\right)}^{n},$$

where $N_{80}=24$ is the number of measurements used for the computation of the trimmed mean. It corresponds to 80% of the observations selected by the 5 min analysis window. The ordered set

(8) $$\left[R_{\left(0\right)}^{n},R_{\left(1\right)}^{n},...,R_{N-1}^{n}\right]$$

is obtained by sorting in ascending order the N=30 absolute speed differences selected at the instant *n* by the analysis window. In this way, (7) is the mean of 80% of the measurements with the smallest absolute value. The trimmed mean is a robust operator [11,12] and is resistant to outliers: anomalous speed differences are removed by sorting and selecting the measurements with the smallest absolute values.

In this paper, a second decision statistic is considered to be a comparison term. The median speed difference has been considered:

(9) $$M\left(nT_{s}\right)=\mathrm{Median}\left(R\left(nT_{s}\right),R\left(\left(n-1\right)T_{s}\right),...,R\left(\left(n-N+1\right)T_{s}\right)\right),$$

where Median(·) is the sample median [11,12].

The behaviour of both decision statistics has been experimentally evaluated using the data collected according to the experimental setup described in Section 4.

The ST regulation mandates to check decision statistic (7) against a decision threshold, $T_{h}$, equal to 10 km/h. If the threshold is passed, a motion conflict error is declared. This threshold is applied in the following to both median and trimmed mean decision statistics.

## 4. Experimental Setup

The decision statistics defined in Section 3 have been experimentally evaluated by using the data collected in two different scenarios. Initially, the experimental campaigns were performed at the Joint Research Centre (JRC) campus in Ispra, Italy. This scenario corresponds to a light-urban environment, where the vehicle’s speeds are constrained by the type of roads present in the JRC campus. Furthermore, experimental data collections were performed in a highway scenario. The experiments involved three vehicles, each one equipped with a multi-constellation GNSS receiver. Thus, three different models of vehicles were used in the experimental setup, which will be named in the rest of the paper as

- Model 1,
- Model 2 and
- Model 3.

These vehicles were equipped with ublox M8T receivers [13] capable of collecting signals from both GPS and Galileo constellations. The use of Galileo is specified by the ST regulation [3]. Moreover, the experiments had the goal of reproducing the operating conditions encountered by a real ST. In addition to the GNSS receivers, the three vehicles were also equipped with OBD data readers. Thus, speed information was continuously collected from the vehicle on-board sensors.

### 4.1. Light-Urban Scenario

In this scenario, the tests were performed using two vehicles (Model 1 and Model 2) and were conducted inside the JRC campus with a closed trajectory being repeated several times. The trajectory selected for the tests is illustrated in [9]. The measurement campaigns were performed during two days and, for each day, several tests of approximately thirty minutes were carried out. The tests performed and the vehicle involved are summarized in Table 1. During the two days, about 4 h of data (8 test runs) were collected. Therefore, since two vehicles were used, 8 h of speed measurements were obtained.

In this scenario, a single-frequency patch antenna was placed on the rooftop of each vehicle and connected to the ublox M8T receiver. Also, a laptop was used to collect data from the ublox M8T receiver and the OBD data reader.

### 4.2. Highway Experiment

**Figure 2:**
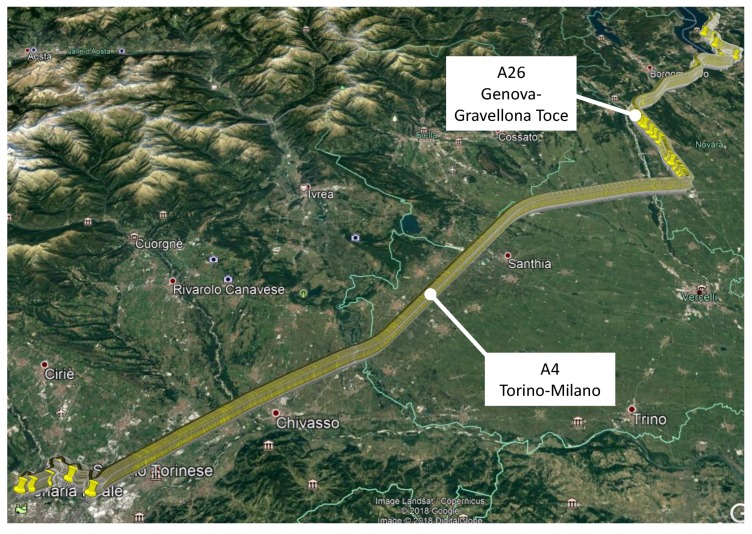
Itinerary followed for the experiment conducted with car Model 3. The tests were conducted in an Italian highway with good visibility conditions.

In the second scenario, vehicle Model 3 was driven on a motorway in Italy under good visibility conditions. This vehicle was also equipped with a ublox M8T receiver, a patch antenna, a laptop and an OBD data reader. In this case, the patch antenna was kept inside the vehicle below the front windshield. Two test campaigns (round-trip journey) were performed under this motorway scenario, as described in Table 1. For each test, the vehicle was driven for approximately 130 km on two Italian motorways. This distance corresponds to 95 km on the A4 (Torino-Milano) motorway and 35 km on the A26 (Genova-Gravellona Toce) motorway, as illustrated in Figure 2.

**Figure 3:**
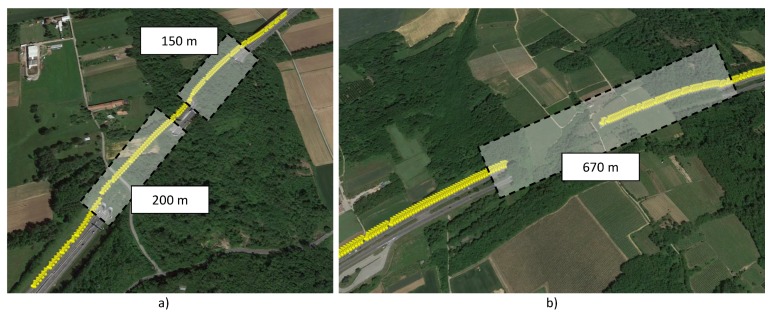
Views of the tunnel sections encountered along the itinerary followed for the tests conducted with car Model 3. (**a**) Short consecutive tunnels; (**b**) Longer tunnel of about 670 m.

In this scenario, the presence of tunnels was noticeable, which had an impact on the GNSS speed computation and, therefore, on the decision statistics described in Section 3. Snapshots showing the presence of two consecutive short tunnels and a long tunnel are shown in Figure 3.

## 5. Experimental Results under Nominal Conditions

This section presents the results obtained by processing the data collected according to the experimental setup described above. The section details results obtained under normal conditions, when no manipulation is applied to GNSS and odometry data. In particular, the resistance of the decision statistics to possible false alarms is experimentally evaluated.

### 5.1. Visibility Analysis

The visibility and Dilution of Precision (DOP) conditions observed during the experiments are briefly analysed here. The analysis shows the benefits of adding Galileo to the navigation solution. In addition to this, the ST regulation suggests to store the signal reception conditions as provided in the NMEA *DOP and Active Satellites* (GSA) sentence [3]:

“*The GPS DOP and active satellites (GSA) command can be used by the VU to determine and record the signal availability and accuracy.*”

The DOP and the number of satellites used in the navigation solution provide an indication of the reception conditions and can be used to determine possible false alarms when poor availability and accuracy are observed. The combined use of Galileo and GPS signals provides increased accuracy and availability conditions reducing the possibility of errors when testing the agreement between GNSS and odometry data.

**Figure 4:**
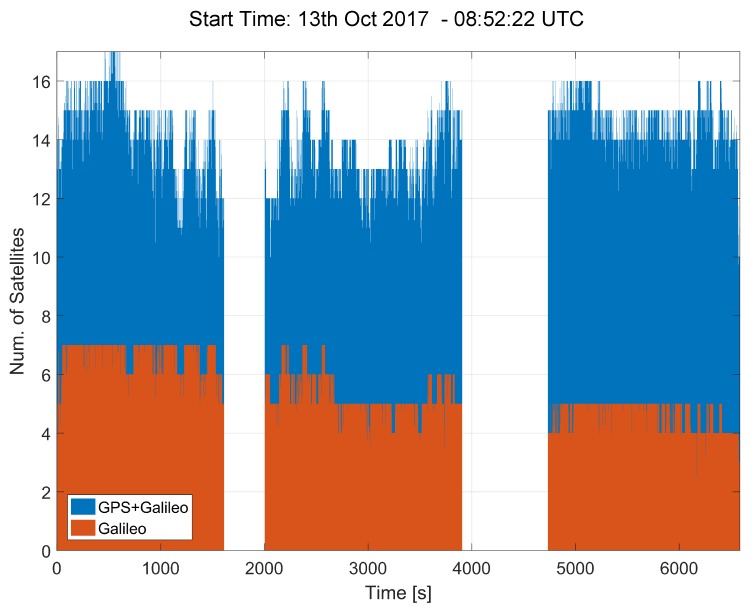
Number of GPS and Galileo satellites used by the ublox receiver for the computation of the navigation solution. Morning 13 October 2017, Model 2 data.

**Figure 5:**
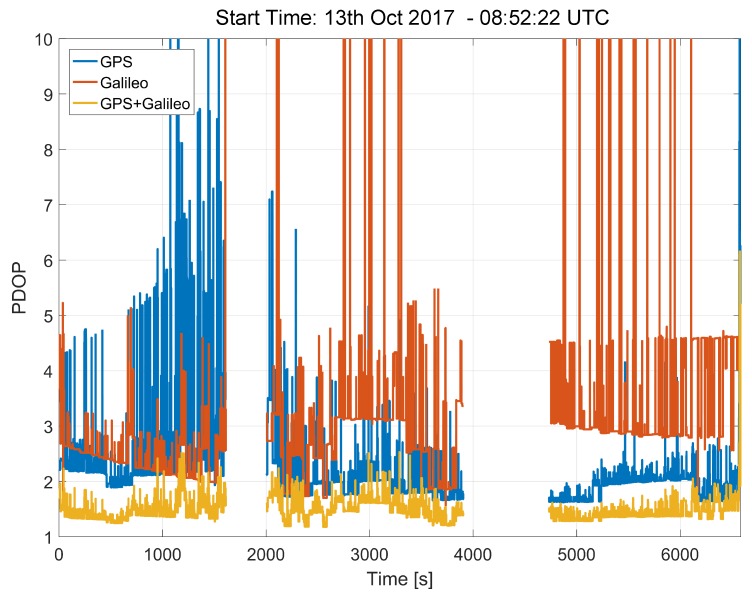
PDOP values obtained considering GPS and Galileo satellites. Morning 13 October 2017, Model 2 data.

Sample availability and DOP plots are shown in Figure 4 and Figure 5, which consider the light-urban scenario for Model 2. This scenario is considered more challenging, in terms of visibility conditions, with respect to the highway experiments. Trees and buildings obstruct the GNSS signal view and thus reduce the number of signals tracked. Since better conditions were experienced for the highway experiments, the related results are not reported here.

The number of satellites used in the navigation solution by the ublox M8T receiver is provided in Figure 4 for the tests conducted on the morning of the 13th October 2017 (tests 6, 7 and 8 in Table 1). The receiver was able to use up to 17 satellites, including a maximum of 7 Galileo satellites. This is considered a very positive result since at least 4 Galileo satellites were always available during the three tests shown in the figure. The tests can be easily identified by the data gaps clearly shown in the figure. The results shown in Figure 4 indicate that it is possible to compute an independent Galileo navigation solution. This independent solution can be used to test the consistency between GPS and Galileo PVT results, potentially providing an additional security layer.

The satellites tracked during the experiments conducted in the morning of the 13 October 2017 led to the Position Dilution of Precision (PDOP) values shown in Figure 5. The addition of the Galileo satellites significantly lower the PDOP. In particular, in the GPS/Galileo combined case, the PDOP is lower than 2 for practically the whole duration of the experiments. This positive result cannot be achieved when considering GPS or Galileo independently.

These results show the benefits of Galileo in terms of visibility and geometry. The combined use of Galileo and GPS improves the effectiveness of the tests prescribed by the ST regulation, providing geometry and visibility conditions that enable the reliable computation of the GNSS speed.

Similar results were obtained when considering Model 1. These results are not repeated here.

### 5.2. Results under Normal Conditions

In the absence of threats, i.e., when the data from the OBD data reader and the GNSS receiver are not falsified, a good agreement between speed measurements is observed. This fact is common to both light-urban and highway scenarios. In this section, the experimental results obtained for the two scenarios are presented. The results detailed here are complementary to those presented in [9]. In particular, reference [9] presented the results obtained during the second set of tests performed on 12 October 2017 (tests 3–5 in Table 1). The results shown in the following were obtained during the last day of tests, on the 13th October. The results obtained for the highway experiments are new and were not presented before.

**Figure 6:**
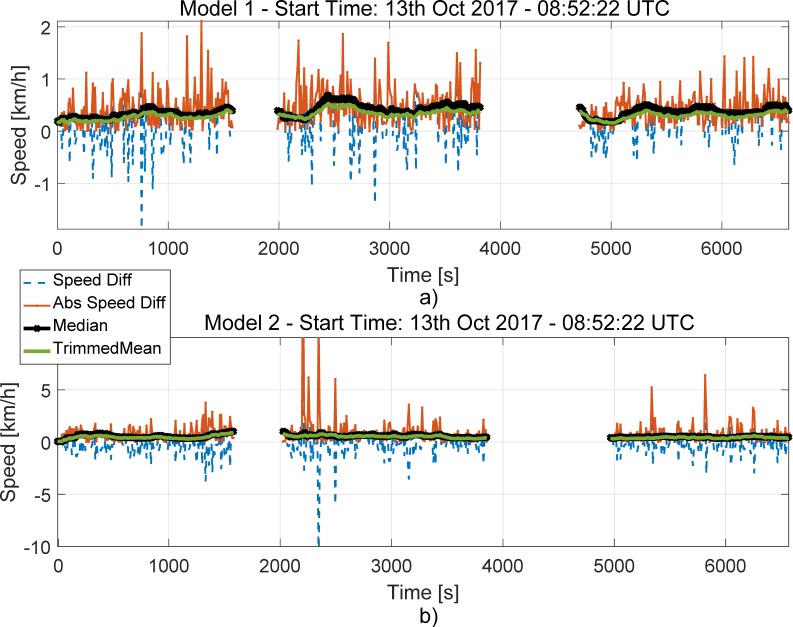
Speed differences and decision statistics as a function of time (start time of the experiment). Morning 13 October 2017. (**a**) Model 1 data; (**b**) Model 2 data.

The speed differences and the decision statistics obtained for the light-urban scenario are shown in Figure 6. The results are provided for tests 6, 7 and 8, which can be clearly identified by the two data gaps visible in the figure. From the figure, it emerges that, for Model 1, the absolute speed differences are below 2 km/h for most of the time. Higher speed differences are observed for Model 2. In particular, discrepancies up to 10 km/h are observed.

However, these differences occur in a sporadic and discontinuous way. These errors are due to small gaps in the OBD data collected for Model 2. These data gaps, which occasionally occurred in the OBD time series, led to the spikes clearly observable in the bottom part of Figure 6. Despite the presence of such spikes, the use of robust operators, such as the trimmed mean and median, makes the decision statistics resistant to this type of phenomena. For both Model 1 and Model 2, the decision statistics are below 1 km/h, which is significantly lower than the 10 km/h threshold selected by the ST regulation. In these experiments, the decision threshold is never passed and no false alarm occurs.

**Figure 7:**
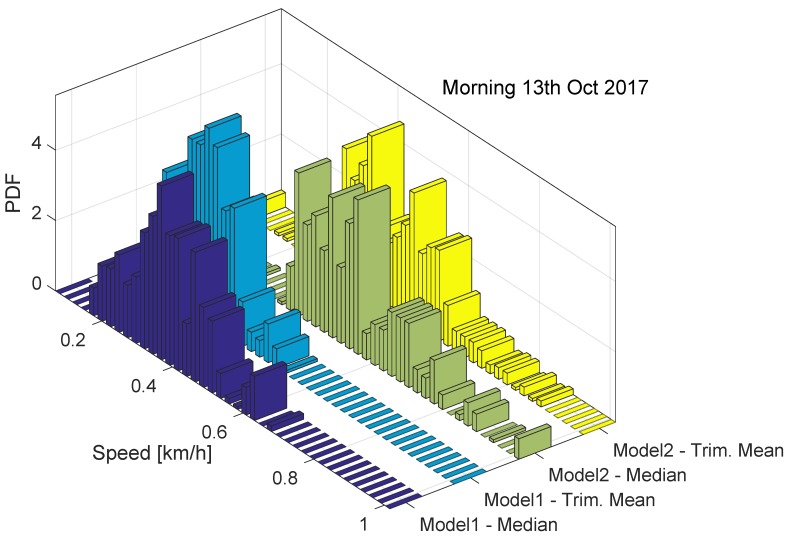
Histograms of the decision statistics prescribed by the ST regulation. Data collected on the morning of the 13 October 2017. Models 1 and 2.

The behaviour of the ST decision statistics is further analysed in Figure 7, which shows the histograms of the decision statistics for the data collected on the 13th October 2017. Both Models 1 and 2 are considered.

The histograms shown in Figure 7 support the findings derived from Figure 6: the decision statistics take values lower than 1 km/h, which is significantly lower that the ST decision threshold. Moreover, slightly lower values are observed for Model 1. This could be due to several factors such as data gaps and different synchronization errors. Despite these effects, the decision statistics are significantly below the decision threshold and robust to different errors. The two decision statistics assume similar values and have a similar behaviour when data are collected in the absence of manipulations.

The results presented in Figure 6 and Figure 7 are consistent with the findings reported in [9]. This shows the repeatability of results obtained.

**Figure 8:**
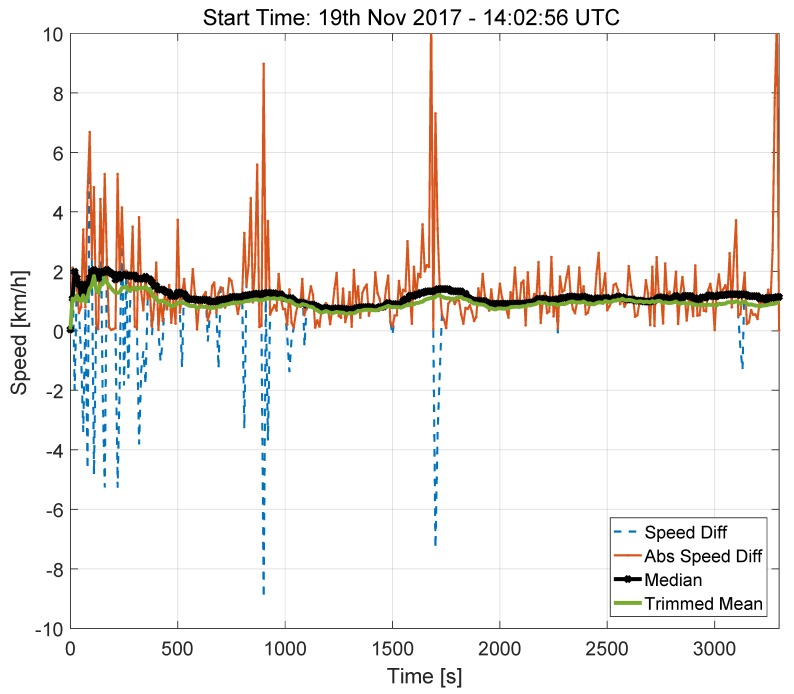
Speed differences and decision statistics as a function of time. Model 3 data, highway test, 19 November 2017. Portion of the highway without tunnels.

The speed differences and the decision statistics evaluated for the highway test are provided in Figure 8. The results obtained for this scenario are similar to that obtained for Model 2 (lower part of Figure 6). Although the test considered in Figure 8 was performed under clear-sky conditions, the speed differences occasionally reach values close to 10 km/h. This result is consistent with the findings obtained for Model 2. It was found that these high values in the speed differences mainly occur when the car is slowing down or stopping.

**Figure 9:**
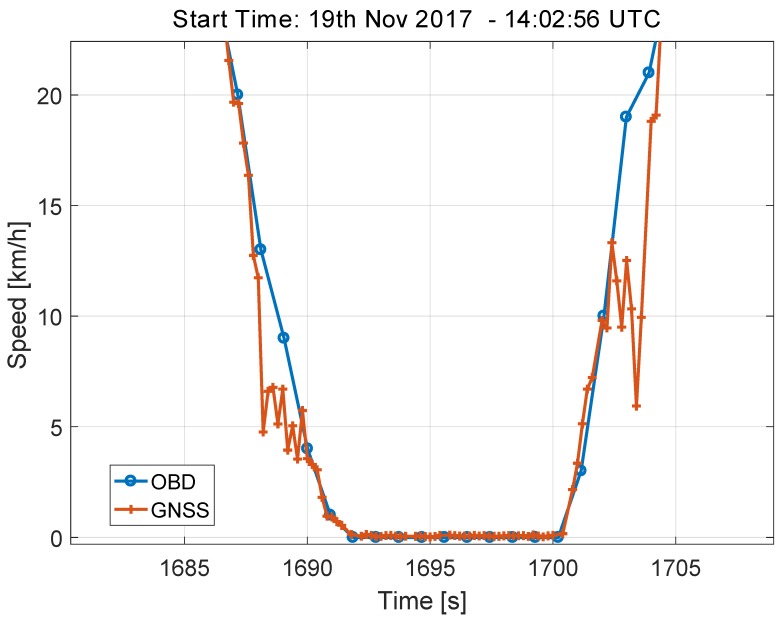
Speed values derived from the OBD and from the GNSS receiver for a small portion of the highway test, where the car is slowing down and then accelerating.

This fact is better analysed in Figure 9, which shows the speed values derived from the OBD and from the GNSS receiver for a small part of the highway test, where the car is slowing down and then accelerating. The data shown in Figure 9 were obtained using the highest data rate supported by the GNSS receiver and with the sampling naturally provided by the OBD data reader. These high sampling rate was adopted here to better analyse the errors in the speed differences. On the contrary, the time series shown in Figure 8 were sampled with a sampling rate equal to 10 s and were obtained using the procedure detailed in Section 2.

From Figure 9, it emerges that the time series from the GNSS receiver has some ripples that causes the high speed discrepancies observed in Figure 8. These errors are consistently observed when the car is performing a speed profile consisting of deceleration and acceleration. Despite these differences, the decision statistics always assume a value significantly lower than the 10 km/h threshold established by the regulation.

As already discussed for the light-urban scenario, this result is due to the robustness of the operators selected for the computation of the decision statistics. Trimmed mean and median effectively remove artefacts arising from the phenomena highlighted in Figure 9.

**Figure 10:**
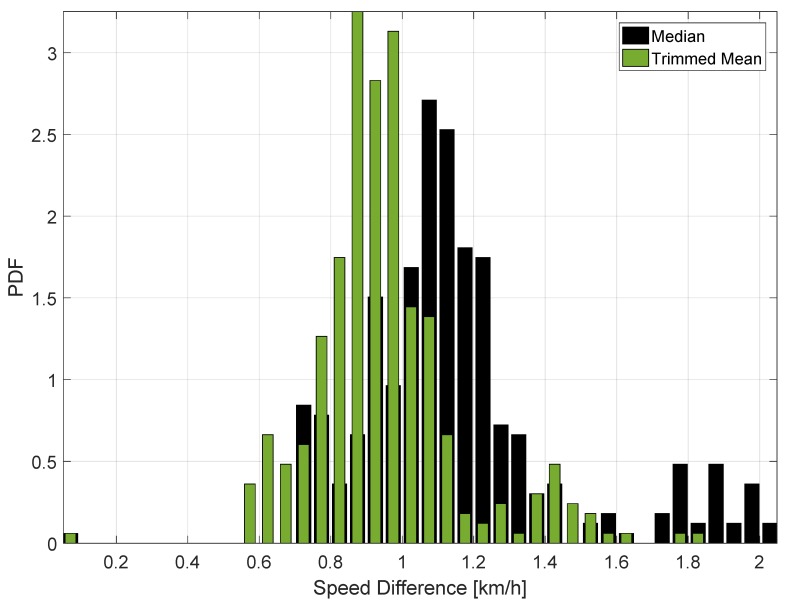
Histograms of the decision statistics prescribed by the ST regulation. Model 3 data, highway test, 19 November 2017.

As for the previous case, the decision statistics obtained in the highway are further analysed in Figure 10, which shows the histograms of the decision statistics. Although, in this case, the values of the decision statistics are slightly higher than those observed in the previous tests, an overall good consistency is observed with previous results. The higher values observed for the decision statistics can be due to the fact the driver was reaching a higher absolute speed. In the highway experiment, the top speed was about 120 km/h.

Despite these differences, the decision statistics evaluated for the highway experiment are always significantly lower than the decision threshold. Also in this case, no false alarm is observed confirming the suitability of the decision statistics defined by the ST regulation.

The results presented above were obtained by considering the data collected on 19 November 2017. The data from 17 November 2017 are analysed in the next section with specific focus on the impact of tunnels.

### 5.3. Impact of Tunnels

As discussed in Section 4, the highway tests conducted on 17 November 2017 involved two tunnel sections. In the first one, two small tunnels, distanced by a clear-sky section of about 50 m, were present. In the second part, a longer tunnel of about 670 m was crossed by the vehicle used for the experiment. When in a tunnel, the GNSS unit is unable to receive GNSS signals and thus is unable to provide a valid position solution. The behaviour of the GNSS receiver and the resulting impact on the ST decision statistics are analysed in the following. The impact of tunnels is further analysed in Section 6 where the duration of the GNSS outage caused by the tunnel is artificially extended to study the breakdown point of the decision statistics.

**Figure 11:**
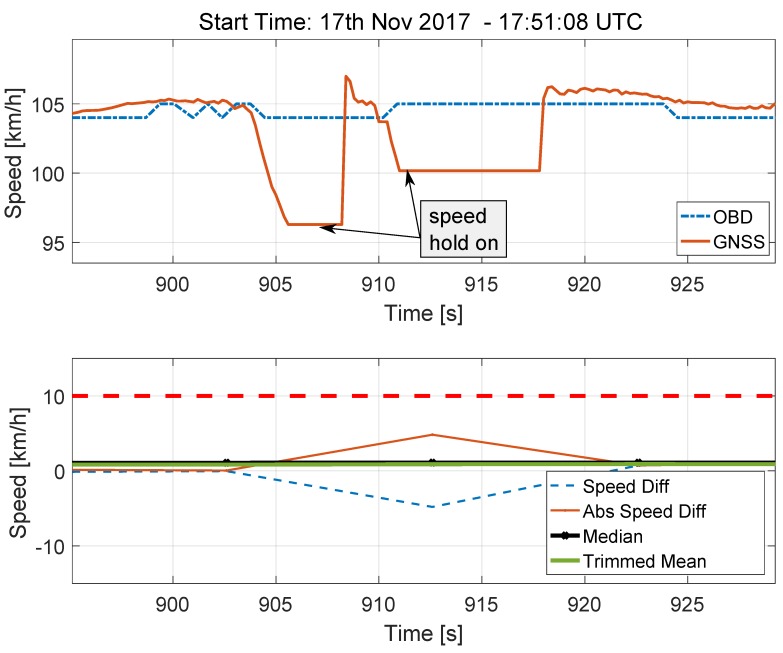
Speed time series (**Upper** part) and decision statistics (**Bottom** part) obtained in the proximity of the two short tunnels shown in Figure 3a.

The speed solutions provided by the GNSS receiver and by the OBD data reader are shown in the upper part of Figure 11 for the case of the two short tunnels. Despite the lack of valid GNSS signals, the receiver still provides speed estimates that are obtained by keeping constant the last valid velocity solution. This fact can be clearly seen in the upper part of Figure 11: the speed estimated in correspondence of the two tunnel sections is constant and separated by a small region, which corresponds to the open-sky portion of the trajectory, where the GNSS receiver reacquires the GNSS signals and provides a valid solution. This portion of the speed solution is indicated as “speed hold on”. The total duration of the two “speed hold on” portions is less than 15 s and has a limited impact on the decision statistics that are analysed in the bottom part of Figure 11.

Since the speed differences are sampled with a time interval of 10 s, only a single outlier is produced. Moreover, maintaining constant the last estimated speed reduces the magnitude of the outlier that is effectively removed by the trimmed mean and median operators. For this reason, the decision statistics are not affected by the presence of the two short tunnels.

**Figure 12:**
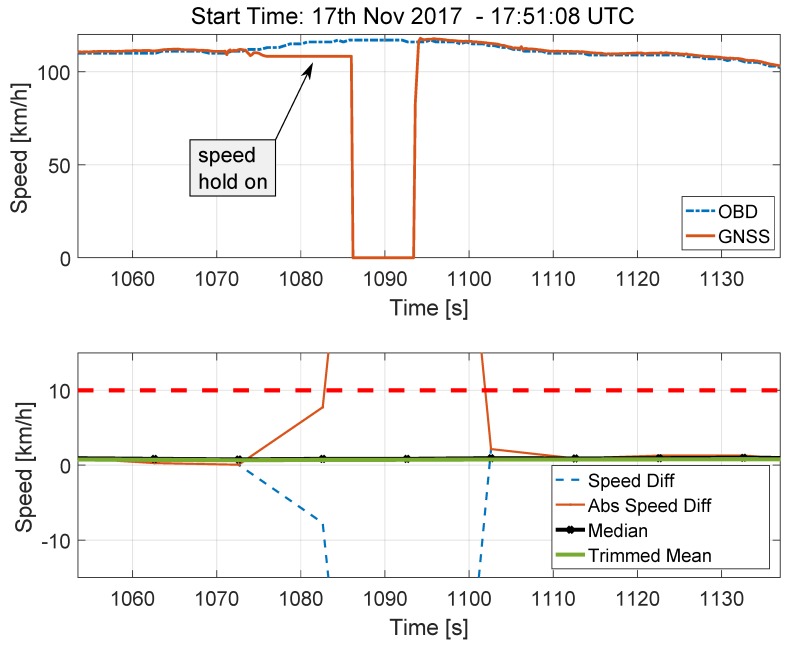
Speed time series (**Upper** part) and decision statistics (**Bottom** part) obtained in the proximity of the tunnel shown in Figure 3b.

The impact of the longer tunnel is analysed in Figure 12. The analysis of the speed time series, which are shown in the upper part of Figure 12, highlights a different behaviour for the GNSS receiver. When a GNSS signal outage longer than that considered in Figure 11 is experienced, the receiver switches between two modes. At first (in the first mode), speed hold on is performed and the last valid speed estimate is kept constant for 10 s. After that (in the second mode), the speed provided by the receiver is set to zero. This phenomenon is clearly visible in the upper part of Figure 12. The total duration of the GNSS signal outage is about 28 s and its impact is clearly visible in the bottom part of Figure 12, which shows the absolute speed differences and the ST decision statistics. Also in this case, the number of outliers caused by the tunnel is too small to impact the decision statistics, showing their robustness against this type of effects.

## 6. Experimental Results under Threats

This section extends the previous results to the case where data manipulation is performed. The effect of losing GNSS signal tracking, for example due to long tunnels, is at first analysed. The goal is to determine the maximum tolerable time for which a vehicle can drive inside a tunnel without breaching the 10 km/h threshold specified in the ST regulations [3]. To do that, the length of the tunnel, shown in Figure 12, is controlled by artificially extending the portion of the GNSS speed time series that is set to zero. This is equivalent to extend the time required to cross the tunnel. During this period, the receiver outputs a speed equal to zero. The data collected from vehicle Model 3 during Test 10 are used in this analysis. Also, note that the duration of the data gap obtained in the original tunnel is approximately 28 s, where the first 10 s are equal to the GNSS speed value held by the receiver and the remaining speed values are set to zero due to loss of tracking.

**Figure 13:**
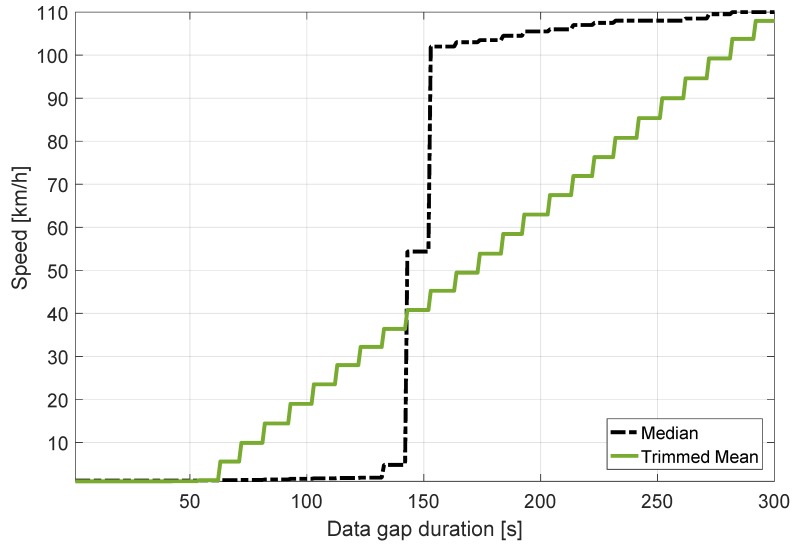
Maxima of the decision statistics as a function of the duration of the data gap applied on the GNSS speed. Highway experiment.

In this context, the decision statistics are computed as a function of the data gap duration, as shown in Figure 13. The results show that the trimmed mean and the median satisfy the 10 km/h condition for data gap durations below 70 and 150 s, respectively. Considering the case of the trimmed mean metric, these results are due to the 20% portion of data that is removed from the computation of the mean. Since 5 min of data are used, the trimmed mean is resistant to data gaps of 60 s (20% of the data duration). When a longer duration is considered, outliers start contaminating the trimmed mean. Since a new measurement is included every 10 s, the breakdown point of the trimmed mean is 70 s: this is the minimum data gap duration that causes biases in the trimmed mean. After this value, the trimmed mean increases linearly until it reaches the maximum speed error value, which is equal to the OBD speed. This linear phenomenon is due to the fact that the number of outliers taken into account increases linearly as the data gap duration increases. The steps, clearly visible in Figure 13 for the trimmed mean, are due to the fact that a new measurement, i.e., a new outlier, is introduced each 10 s.

The median estimator has a breakdown point higher than that of the trimmed mean. It corresponds to 50% of the data used for the computation of the sample median [11]. For this reason, the data gap duration necessary to significantly bias the median decision statistics is 150 s. The median rejects all the outliers (those corresponding to the zero GNSS speed values) when their number is less than the number of inliers. In contrast, when the number of outliers is larger than that of the inliers, the median speed error converges to the median of the outliers. Since the GNSS speed is equal to zero, this value is equal to the speed registered by the OBD data reader. For this reason, a sharp transition is observed for the curve in Figure 13 related to the median operator.

The transition state of this step function is measured at approximately 150 s. Also, the quantization behaviour of these speed error time series is noticeable due to the fact that the decision statistics were computed every 10 s according to the ST regulations [3].

Subsequently, the impact caused by synchronization errors between the GNSS and OBD data has been analysed by introducing data delays between the two speed time series. The goal of this test was to determine the maximum latency values that can be tolerated by the system without triggering false alarms. In addition, for large delay values, this experimental test can be considered as a simplistic form of meaconing or replay attack [6].

**Figure 14:**
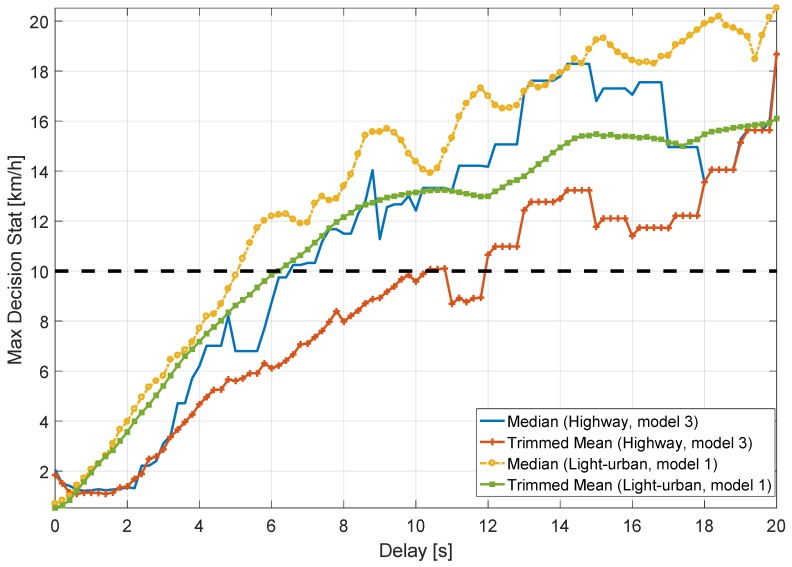
Maxima of the decision statistics as a function of the delay between GNSS and odometry data. Highway and light-urban experiments.

The data collected were progressively shifted in time producing a latency between GNSS and OBD speeds. Then, the absolute speed differences were obtained and both median and trimmed mean decision statistics were computed. For this analysis, the data collected during Test 6 with vehicle Model 1 and Test 10 with vehicle Model 3 were employed for the light-urban and highway scenarios, respectively. Figure 14 shows the maxima of the median and trimmed mean decision statistics as a function of the relative delay between the speeds from the two sensors for the two selected scenarios. Overall, the maxima of the two decision statistics increase as the relative delay increases for both scenarios. Also, it can be observed that, for both environments, the median is always above the trimmed mean and for all the cases the 10 km/h threshold is only passed when the relative delay is above 5 s. The trimmed mean passes this threshold after approximately 6 and 10 s in the light-urban and highway environments, respectively. Results also show that tolerable delays are larger when the vehicles are driving in a highway with respect to the light-urban scenario. This is due to the fact that, in a highway scenario, the vehicles move at a more constant speed. This low speed variability increases robustness towards synchronization errors. In addition, the median surpasses the 10 km/h threshold when the synchronization delay is larger than 5 s for the light-urban scenario and 6 s for the highway environment. Two conclusions can be drawn from these results. The first one is that the decision statistics selected for detecting a motion conflict can tolerate a significant latency (synchronization error) between GNSS and odometry data. The second conclusion is that a simple meaconing attack seems unlikely to be successful. Indeed, the decision statistics mandated by [3] are able to detect inconsistencies between motion data even when only short delays are introduced. When GNSS and odometry data come from different and unrelated scenarios, the decision statistics reach higher values than those shown in Figure 14.

For this reason, a meaconing attack can be successful only if both sensors are compromised.

**Figure 15:**
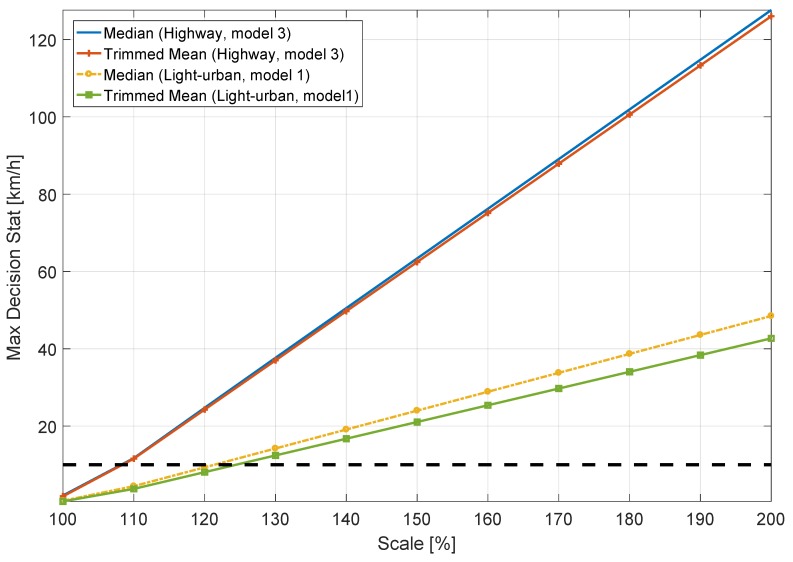
Maxima of the decision statistics as a function of the speed scaling applied to the odometry data. Highway and light-urban experiments.

Finally, the scenario where the data collected using the OBD sensor is scaled with respect to the GNSS data is examined. More specifically, the speed from the OBD sensor is multiplied by a constant that is used to change its value. In this way, a lower speed is recorded from the OBD data reader. The goal of this analysis is to reflect the infringement situation where the driver of the vehicle intends to speed up and, simultaneously, to alter the OBD data in order to record a lower speed. As a consequence of this action, the same speed profile is obtained but with different overall values. Thus, the maxima of the decision statistics as a function of the speed scaling factor are provided in Figure 15 for the aforementioned scenarios. Results show that when the GNSS speed is approximately 30% lower than the odometry data, the decision threshold is passed by both decision statistics for the light-urban scenario, whereas only a 10% scaling is needed to cross the threshold when the vehicle is driving on a highway. These phenomena are expected since the average speed of the experiment carried out in the light-urban scenario is about 33 km/h and, when there is a scale difference of about 30%, an absolute error of about 10 km/h is observed. Furthermore, the average speed of Model 3 on the highway test was 110 km/h producing a 10% margin before passing the threshold value. These results indicate that the decision threshold allows a driver to over-speed only by an amount equal to 10 km/h. Thus, the figure confirms the ability of the two decision statistics to detect discrepancies between speed data above the 10 km/h threshold.

## 7. Conclusions

In this work, the decision statistics, introduced by the ST regulation to verify the consistency of speed data from different sensors, have been experimentally characterized using three vehicles and considering different scenarios. In this respect, both light-urban and highway environments were analysed. These scenarios were selected as they were considered the most representative environments where a truck driver is supposed to operate. Under heavy urban conditions, the presence of buildings and other obstacles will likely degrade the performance of the GNSS receiver. On the other hand, the less predictable behaviour of the driver, due to harsher traffic and road conditions, will introduce stronger signatures in the speed profile. The analysis of these effects is left for future work. Specific focus was devoted to synchronization aspects, and possible latencies between GNSS and odometry data were considered.

The experimental analysis showed that the test statistics are resilient to false alarms, for example caused by synchronization errors. This fact is due to the inherent robustness of the operators adopted for the definition of the test statistics. In both light-urban and highway experiments, no false alarm was recorded. This is also due to the decision threshold of 10 km/h specified in regulation [3], which provides sufficient margin against false alarms.

In the highway scenarios, the presence of tunnels was also considered. For short tunnels, the ST decision statistics are only marginally impacted by the lack of valid GNSS measurements. The length of the tunnels was artificially extended by setting to zero the speed provided by the GNSS receiver. This analysis was performed to study the ability of the decision statistics to withstand data gaps. The trimmed mean test statistics is able to operate with data gaps up to 60 s before being significantly biased. The median test statistics has a better breakdown point and it is able to provide unbiased estimates for GNSS data outages up to 150 s.

The test statistics are also resistant to latencies between GNSS and odometry data. In the light-urban scenario, which has a less constant speed profile than the highway environment, latencies up to 5 s are tolerated. These values are sufficient to protect the decision statistics against false alarms originating from synchronization errors and, at the same time, make meaconing attacks unfeasible. In this respect, an attacker has to falsify the data from both odometer and GNSS at the same time and in a consistent way. It was shown that the decision statistics are effective against data manipulation forms such as data scaling: only discrepancies lower than the 10 km/h threshold are undetected by the tests prescribed by the ST regulation.

The paper also showed the level of maturity of the Galileo system and the benefits of using two GNSS constellations for reliable velocity estimation. Improved performance is expected when the full Galileo constellation will be available. The results obtained are encouraging and show the effectiveness of the detection mechanisms prescribed by the ST regulations. Additional experiments should be performed to confirm the validity of the findings described. In particular, additional scenarios and different receiver types should be considered. 

## Figures and Tables

**Table 1:** Summary of the tests performed using different vehicles.

Date	Test Name	Cars
12 October 2017 Morning	Test 1	Model 1 + Model 2
	Test 2	Model 1 + Model 2
12 October 2017 Afternoon	Test 3	Model 1 + Model 2
	Test 4	Model 1 + Model 2
	Test 5	Model 1 + Model 2
13 October 2017 Morning	Test 6	Model 1 + Model 2
	Test 7	Model 1 + Model 2
	Test 8	Model 1 + Model 2
17 November 2017	Test 9	Model 3
19 November 2017	Test 10	Model 3
